# STAT3 and its targeting inhibitors in osteosarcoma

**DOI:** 10.1111/cpr.12974

**Published:** 2020-12-31

**Authors:** Yun Liu, Shijie Liao, Samuel Bennett, Haijun Tang, Dezhi Song, David Wood, Xinli Zhan, Jiake Xu

**Affiliations:** ^1^ Department of Spine and Osteopathic Surgery First Affiliated Hospital of Guangxi Medical University Nanning Guangxi China; ^2^ Division of Regenerative Biology School of Biomedical Sciences University of Western Australia Perth WA Australia; ^3^ Research Centre for Regenerative Medicine Guangxi Key Laboratory of Regenerative Medicine Guangxi Medical University Nanning Guangxi China; ^4^ Department of Trauma Orthopedic and Hand Surgery First Affiliated Hospital of Guangxi Medical University Nanning Guangxi China; ^5^ Department of Orthopedic Guangxi hospital for nationalities Nanning Guangxi China

**Keywords:** metastasis, oncogenes, osteosarcoma (OS), signal transducer and activator of transcription 3 (STAT3), signalling, STAT3 inhibitor

## Abstract

Signal transducer and activator of transcription 3 (STAT3) is one of seven STAT family members involved with the regulation of cellular growth, differentiation and survival. STAT proteins are conserved among eukaryotes and are important for biological functions of embryogenesis, immunity, haematopoiesis and cell migration. STAT3 is widely expressed and located in the cytoplasm in an inactive form. STAT3 is rapidly and transiently activated by tyrosine phosphorylation by a range of signalling pathways, including cytokines from the IL‐6 family and growth factors, such as EGF and PDGF. STAT3 activation and subsequent dimer formation initiates nuclear translocation of STAT3 for the regulation of target gene transcription. Four STAT3 isoforms have been identified, which have distinct biological functions. STAT3 is considered a proto‐oncogene and constitutive activation of STAT3 is implicated in the development of various cancers, including multiple myeloma, leukaemia and lymphomas. In this review, we focus on recent progress on STAT3 and osteosarcoma (OS). Notably, STAT3 is overexpressed and associated with the poor prognosis of OS. Constitutive activation of STAT3 in OS appears to upregulate the expression of target oncogenes, leading to OS cell transformation, proliferation, tumour formation, invasion, metastasis, immune evasion and drug resistance. Taken together, STAT3 is a target for cancer therapy, and STAT3 inhibitors represent potential therapeutic candidates for the treatment of OS.

## Graphical abstract

Signal transducer and activator of transcription 3 (STAT3) is a member of the STAT protein family, vitally important for eukaryotic cells. We review the molecular structure and function of STAT3 and its isoforms, highlighting signalling pathways for the regulation of gene transcription. A critical appraisal of STAT3 in cancers, such as osteosarcoma, is provided emphasizing potential therapeutic approaches targeting STAT3 and its inhibitors

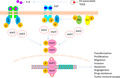

**Abbreviations**
DDR1: discoid domain receptor 1
GM‐CSF: granulocyte‐macrophage colony‐stimulating factor
GSEA: gene set enrichment analysis
lncRNA: long non‐coding RNA
miRNA: micro‐RNA
MMP: matrix metalloproteinase
PDGF(R): platelet‐derived growth factor (receptor)
PI3K: phosphatidylinositol‐4,5‐bisphosphate 3‐kinase
STK35: serine/threonine kinase 35
VEGF(R): vascular endothelial growth factor (receptor

## 1 INTRODUCTION

Signal transducers and activators of transcription (STAT) proteins are latent cytoplasmic transcription factors that are activated by cytokines and growth factors.^1^ Activated STATs translocate to the nucleus where they bind to promoter DNA elements and regulate gene transcription.^2^ Seven STAT family members have been discovered in human and mouse: STAT1, STAT2, STAT3, STAT4, STAT5A, STAT5B and STAT6.^3^ STATs are cell signalling transducers for vital biological functions of cell growth, differentiation and survival.^4^ STATs are conserved among eukaryotes and are involved with a wide range of functions including embryogenesis, immunity, inflammation, haematopoiesis and cell migration.^4^ STAT3 is widely expressed and is transiently activated in response to epidermal growth factor (EGF) and interleukin‐6 (IL‐6) by tyrosine phosphorylation.^5^, ^6^ STAT 3 plays a crucial role in mediating cell growth, differentiation and survival signals of the IL‐6 cytokine family via the gp130 receptor subunit.^4^, ^7^ STAT3 gene disruption leads to embryonic lethality in the mouse, indicating the vital role of STAT3 for mammalian development.^8^ STAT3 is constitutively activated during the onset and progression of a variety of cancers, including multiple myeloma, leukaemia, lymphomas and solid tumours.^9^ STAT3 overexpression is implicated in the development, progression and poor prognosis of osteosarcoma (OS) and emerges as a potential therapeutic target for the treatment of OS.^10^, ^11^, ^12^ OS is the most common form of primary bone malignancy and the eighth most common childhood cancer, affecting approximately 2.4% of all childhood cancers.^13^ OS has a bimodal age distribution with peaks during adolescence (10‐14 years) and for adults aged over 65 years.^13^ During adulthood, OS may occur as a second malignancy related to Paget's disease.^13^ OS is thought to be derived from osteogenic progenitor mesenchymal or committed osteoblast precursor cells.^13^, ^14^ The 5‐year survival rate for the treatment of OS is estimated to be 60%‐70%, and poor prognosis depends on factors including the rate of metastases and chemotherapeutic resistance.^13^, ^15^ Here, we review the structure and function of STAT3, the role of STAT3 in OS and STAT3 inhibitors for the treatment of OS.

## 2 DOMAIN STRUCTURE AND BIOLOGICAL FUNCTION OF STAT3

### 2.1 Structure of STAT3

**FIGURE 1:**
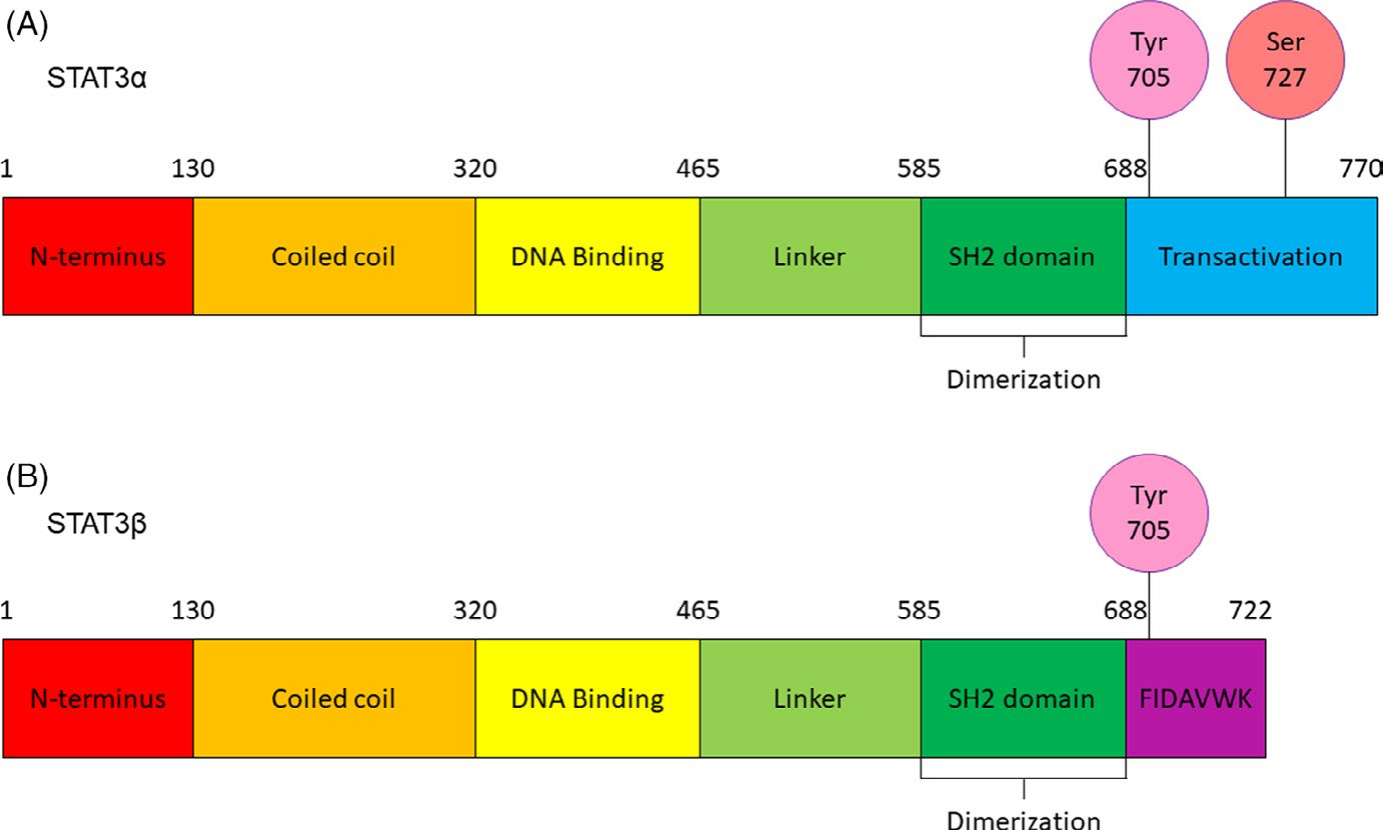
STAT3 secondary structure. STAT3 protein structure includes N‐terminal domain, coiled coil and DNA binding domains, a linker domain, SH2 domain involved with dimer formation and the C‐terminus. The C‐terminus of the (A) STAT3α isoform and (B) STAT3β isoform confers distinct functions of the two isoforms. STAT3, signal transducer and activator of transcription 3

**FIGURE 2:**
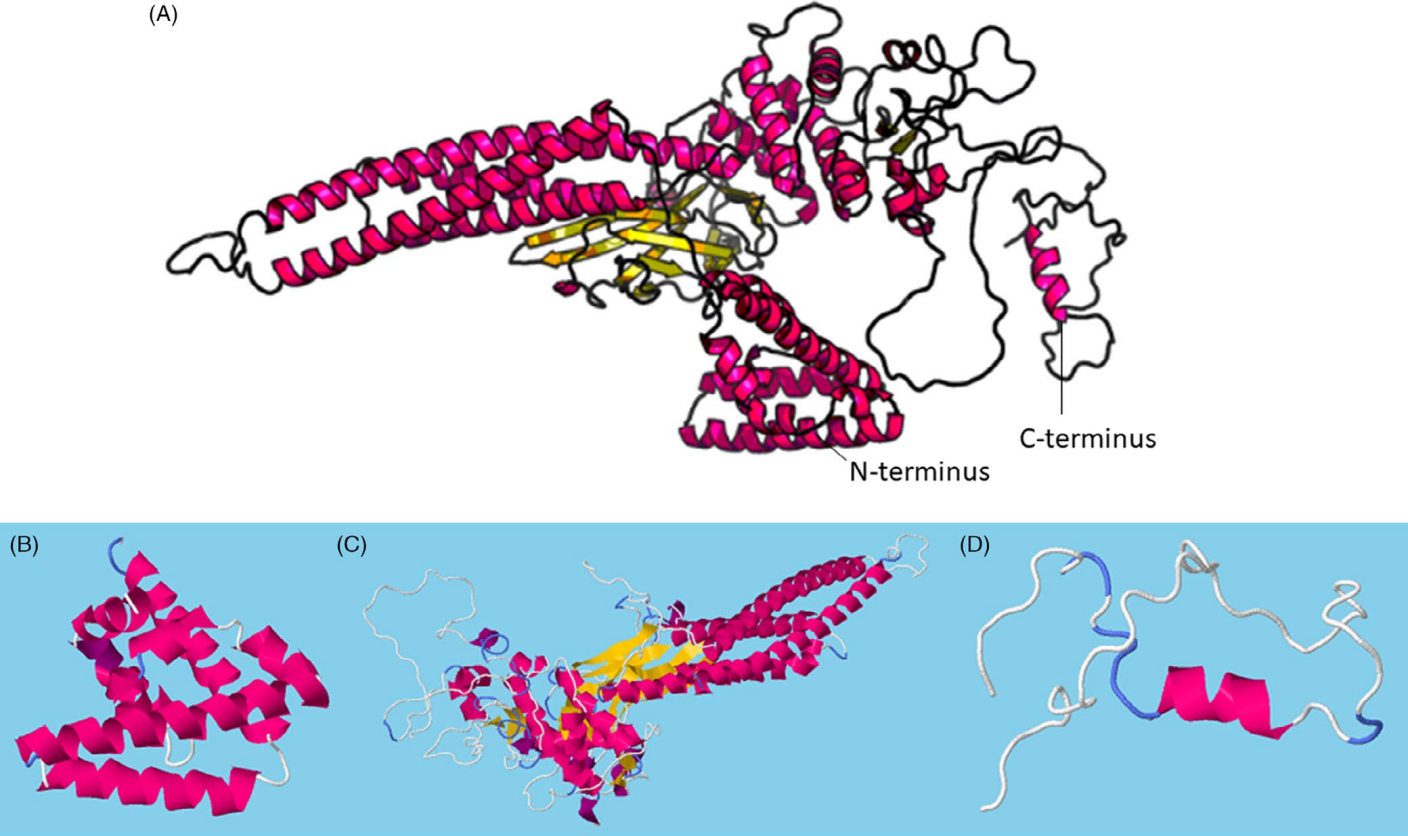
STAT3α isoform tertiary structure predicted by RaptorX template‐based protein structure modelling bioinformatics analysis, http://raptorx.uchicago.edu/StructPredV2/predict/. (A) Whole sequence predicted model. (B) N‐terminal domain, aa1‐124. (C) Coiled coil, DNA binding, linker and SH2 domains, aa125‐715. (D) C‐terminal transactivation domain, aa716‐770. STAT3, signal transducer and activator of transcription 3

The human STAT3 gene is located on chromosome 17 (17q21.2) and has 24 exons.^16^ The STAT3 protein was originally described as acute‐phase response factor (APRF) and consists of six domains: an amino‐terminus, a coiled‐coil domain, the DNA binding domain, a linker domain, the Src Homology 2 (SH2) domain and a carboxy‐terminal transactivation domain.^17^, ^18^ Four STAT3 isoforms (α, β, γ and δ) have been identified.^19^ The STAT3γ (72kDa) and STAT3δ (64kDa) isoforms are produced by proteolytic processing, and they appear to play an important role in the regulation of granulocyte development.^19^ The STAT3α and STAT3β isoforms are produced by alternative splicing of exon 23 and have distinct biological functions.^16^, ^20^, ^21^ Both STAT3α and STAT3β contain a tyrosine phosphorylation activation site (Y705) and SH2 domain within the C‐terminus^22^ (Figure 1A,B). STAT3 activation by phosphorylation of Y705 leads to the formation of homo‐ or heterodimers via the SH2 domain and nuclear translocation for the regulation of gene transcription.^5^, ^23^ STAT3α (92kDa) contains two important phosphorylation sites (Y705 and S727) within the C‐terminus (Figure 1A).^20^ STAT3 activation of transcription is maximal with dual phosphorylation of Y705 and S727.^24^ STAT3α transcriptional activation of target genes may involve the recruitment of co‐factors, such as CREB‐binding protein (CBP)/p300, via the C‐terminal transactivation domain.^25^ STAT3β (83 kDa) is produced by alternative splicing, which results in frameshift coding for a truncated C‐terminus lacking 55 amino acids and S727, which are replaced by seven amino acids and a stop codon^26^ (Figure 1B). STAT3α and STAT3β functional differences appear to be due to the presence or absence of the acidic C‐terminal tail of STAT3α.^22^ STAT3α appears to have greater transcriptional activity than STAT3β.^22^ STAT3β appears to have a greater potential for constitutive activity, to bind DNA with greater affinity and to form more stable dimers than STAT3α.^22^, ^23^ The STAT3α acidic C‐terminal tail is thought to destabilize the active dimeric form and DNA binding of STAT3α, resulting in rapid dephosphorylation.^22^ The STAT3α acidic C‐terminal tail represents a potential mechanistic target for STAT3 deactivation. STAT proteins have distinct functional domains: the 130aa N‐terminal domain mediates cooperative binding to multiple DNA sites, DNA binding specificity is conferred by residues 400‐500 residues of the SH2 domain participate in dimer formation and the C‐terminus is involved with the activation of transcription.^24^, ^27^, ^28^, ^29^, ^30^ Dimerized STAT proteins are generally thought to bind to DNA target sites via a 9‐bp consensus sequence, TTCCGGGAA.^29^ STAT3 tertiary structure may be considered in three domains and has distinct functional elements (Figure 2A‐D).^31^, ^32^ STAT3 alpha‐helix 2 of the coiled–coil domain sequence element, R214/215, was shown to be required for nuclear translocation and subsequent export of STAT3.^33^, ^34^ STAT3 R214/215 appears to be the importin alpha5 binding site for nuclear translocation STAT3.^35^ DNA binding sequence element, R414/417, is also required for nuclear translocation by stabilizing the STAT3 dimer for importin binding.^33^, ^35^ The STAT3 N‐terminal and SH2 domains are potential targets for cancer therapy. The STAT3 SH2 domain contains three subpockets which represent potential therapeutic targets: the Y705‐binding pocket, an L706‐subsite and a unique STAT3 hydrophobic side pocket.^36^ The STAT3 N‐terminal domain is vital for function and contains a 4‐helix bundle that is a potential target for cancer therapy.^29^, ^37^ STAT3 helix 2 analogs were rationally designed and demonstrated the potential to induce apoptosis of breast cancer cells.^37^ Further research of STAT3 structural properties will improve the potential for disease‐specific targeted therapeutic applications.

### 2.2 Biological function of STAT3

**FIGURE 3:**
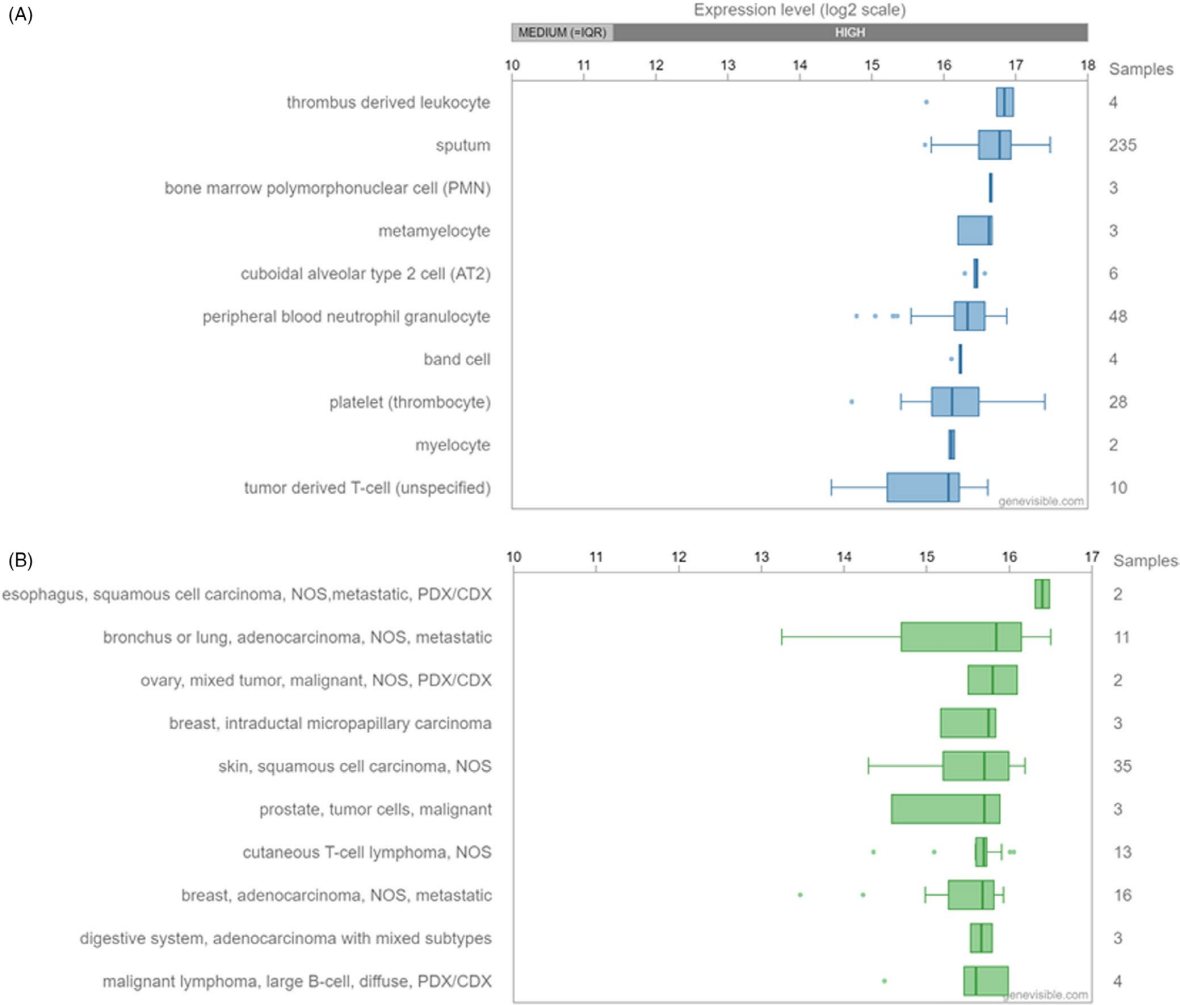
(A) STAT3 expression analysis performed by Genevisible®, https://genevisible.com/search. STAT3 expression analysis across 518 tissues, showing the 10 most highly expressed, including leucocyte and T‐cell populations. (B) STAT3 expression analysis across 539 cancers, showing the 10 most highly expressed, including metastatic lung, breast and adenocarcinomas. STAT3, signal transducer and activator of transcription 3

STAT3 is widely expressed and plays a vital role during mammalian development.^6^, ^8^ Expression analysis performed by Genevisible® across over 500 human tissues indicates leucocyte and T‐cell populations are highly expressive of STAT3 (Figure 3A).^38^ Recent research indicates STAT3 protein is expressed in CD4^+^ T cells, T helper Th17 cells, Th1 and Th2 cells and the STAT3α isoform might interact with proteins, such as Prohibitin 1, for modulation of pathological immune responses.^39^ STAT3 was originally identified as APRF for its role in mediating the acute‐phase response in liver.^17^ STAT3 was found to be rapidly activated by IL‐6 for the regulation of acute‐phase gene transcription.^17^ STAT3 was subsequently found to be activated by the family of cytokines acting via the gp130 receptor subunit, including IL‐6, leukaemia inhibitory factor (LIF), oncostatin M, IL‐11 and ciliary neurotropic factor.^7^ STAT3 is also activated by growth factors, such as EGF and platelet‐derived growth factor (PDGF) which signal via tyrosine kinase receptors.^5^, ^40^ STAT3 plays a central role in JAK/STAT signal transduction for the regulation of cell growth, differentiation and survival.^4^ STAT3 is involved in biological processes of the immune response, inflammation and haematopoiesis.^4^ STAT3 is rapidly and transiently activated by tyrosine phosphorylation.^5^ STAT activation is physiologically regulated by mechanisms including negative feedback via cytokine inducible SH2 proteins and suppressor of cytokine signalling (SOCS) proteins, blocking of STAT DNA binding in the nucleus by protein inhibitor of activated STAT, and STAT deactivation by tyrosine phosphatases, such as TC45.^41^, ^42^ STAT3 sequence element, R214/215, was shown to be involved in the regulation of nuclear transport of STAT3.^34^ STAT3‐mediated signalling appears to be negatively regulated by low molecular weight‐dual specificity phosphatase two (LMW‐DSP2) in vitro and in vivo, resulting in decreased nuclear translocation of STAT3.^43^ Dysregulated or constitutive activation of STAT3 may lead to adverse functional effects, such as impaired immunity and the development of inflammatory disease and cancers.^9^, ^44^, ^45^ Further research is needed to investigate cellular and molecular signalling mechanisms regulating the activity of STAT3

STAT3 activation is regulated by a complex network of signalling pathways, and its specificity is incompletely understood. STAT3 is activated by tyrosine kinase phosphorylation in response to cytokines and growth factors, such as IL‐6, EGF and PDGF.^7^, ^40^, ^46^ STAT3 is classically activated by cytokines via the Janus kinase‐STAT (JAK‐STAT) pathway involving JAK1‐ and JAK2‐mediated tyrosine phosphorylation.^47^ JAK‐STAT signalling pathways are conserved in eukaryotes and are regulated by intrinsic and extrinsic stimuli, which enable cells and tissues to respond to environmental changes.^47^ STAT3 activation by PDGF is mediated by JAKs (JAK1, JAK2 and Tyk2), independently of the presence of any single JAK.^48^ STAT3 is activated by EGF and IL‐6 and, unlike other STATs, is not activated by interferon‐γ.^5^ STAT3 activation by IL‐6 may be prolonged by binding of the IL‐6 and EGF receptors, which may lead to constitutive STAT3 activity associated with disease.^49^ STAT3 is activated by non‐receptor tyrosine kinases, such as Src and Abl, via signalling pathways which are required for normal mitogenesis and may lead to oncogenic transformation.^50^ The pathway of STAT3 activation by EGF effected by JAKs may depend on upstream Src kinase signalling.^46^ STAT3 may be activated as a downstream effector of heterotrimeric guanine nucleotide‐binding proteins (G proteins) signalling.^51^ STAT3, together with ERK, activation is required during the Toll‐like receptor–induced IL‐10 production by B cells.^52^ Taken together, STAT3 is activated both as a specific target and a downstream effector by a complex network of cellular signalling pathways to perform a wide range of biological functions. The disease‐specific role of STAT3 activated signalling in the immune response, inflammatory disease and cancer warrants further investigation.

STAT3 appears to play an important role in mediating cellular differentiation via its isoforms.^19^ The STAT3 isoforms (α, β, γ and δ) are selectively expressed and activated during the regulation of granulocyte differentiation in vitro.^19^ The ratio of STAT3 isoforms increased towards STAT3β with granulocytic differentiation and maturation.^19^ The STAT3α and STAT3β isoforms are produced by alternative splicing and differ structurally and functionally.^16^, ^22^ STAT3α ablation results in postnatal death of mice in vivo.^20^ STAT3β function can rescue embryonic lethality of total STAT3 deletion in vivo, and STAT3β‐depleted mice are viable and fertile.^20^ Both STAT3α and STAT3β exert an anti‐inflammatory function, and their role in inflammation is complex.^20^ STAT3α and STAT3β are widely co‐expressed, and the intracellular ratio of STAT3α to STAT3 is modulated by external stimuli.^19^, ^26^ The ratio of STAT3β to STAT3α is increased in response to bacterial endotoxin lipopolysaccharide (LPS) in vivo, and STAT3β plays an important role in the control of systemic inflammation in vivo.^21^ The STAT3α and STAT3β isoforms appear to indicate the level at which granulocyte colony‐stimulating factor (G‐CSF) signalling diverges from immature normal to leukaemic human myeloid cells in vitro.^53^ The ratio of STAT3α and STAT3β isoforms may affect the G‐CSF‐induced differentiation of myeloid cells.^53^ The cellular signalling pathways of STAT3 isoforms remain largely unknown. Further investigation of the role of STAT3 isoforms in mediating cellular genetic programming in response to disease‐specific external stimuli is required.

## 3 THE ROLE OF STAT3 IN CANCERS AND OS

STATs are important mediators of cell signalling for a wide range of biological functions including cell growth, differentiation and survival events of immunity and inflammation. STATs may be activated by oncoproteins and contribute to the process of malignant transformation by promoting cell proliferation and preventing apoptosis.^9^ Dysregulated activation of STATs is frequently observed in human cancers. STAT3 constitutive activation is evident during the onset and progression of various cancers, including multiple myeloma, leukaemia, lymphomas and solid tumours.^9^ Expression analysis by Genevisible® across over 500 human cancers indicates that STAT3 is highly expressed in metastatic carcinomas such as lung, breast and adenocarcinoma (Figure 3B).^38^ STAT3 dysregulation is thought to be involved with tumour progression, angiogenesis and metastasis. STAT3 appears to play a crucial role in the development of OS and represents a biomarker, prognostic indicator and a potential molecular target for OS gene therapy.^10^ Further research is needed to investigate STAT3 as a predictive biomarker in prognosis and potential clinical therapeutic applications for cancers.

### 3.1 The oncogenic potential of STAT3 in OS

STAT3 is implicated in the onset and progression of human cancers including multiple myeloma, leukaemia, lymphomas and solid tumours.^9^ STAT3 overexpression is observed and associated with poor prognosis of solid tumours, such as gastric cancer, lung cancer, gliomas, hepatic cancers, OS, prostate cancer and pancreatic cancer.^54^ STAT3 overexpression is significantly associated with poor prognosis of solid tumours, including OS, for three‐ and five‐year overall survival.^54^ OS is the most common primary malignancy of bone and the eighth most common childhood cancer.^13^ OS may occur during adulthood as a second malignancy of Paget's disease.^13^ The overall five‐year survival rate of OS is estimated to be 68%.^13^ STAT3 activation is abnormal in human cancers, such as OS, and transformed cell lines indicating the potential role of STAT3 in oncogenesis.^50^, ^55^ STAT3 was demonstrated as a proto‐oncogene by the potential to mediate cellular transformation in vitro.^56^ STAT3 constitutive activation directly by oncoprotein, Src, was shown to be a crucial signalling pathway for cell transformation in vitro.^57^, ^58^ Interestingly, the STAT3β isoform appears to abrogate gene induction by Src and to block cell transformation in a pathway‐specific manner.^58^ STAT3 overexpression is evident in OS tissues and OS cell lines.^10^, ^59^, ^60^ STAT3 mRNA and protein expression levels are significantly higher in OS tissues compared to normal bone or chondroma tissues and OS cell lines.^10^, ^59^, ^60^ STAT3 expression is increased in multidrug resistant (MDR) OS cell lines and is a predictive marker for poor response to chemotherapy for OS.^59^, ^61^ STAT3 is upregulated as a downstream target of Notch genes (Notch 1, 2 and 4) in highly metastatic murine OS K7M2 cells.^62^ The role of STAT3 in OS stem cells (OSS) is largely unknown. Putative OSS markers include aldehyde dehydrogenase, CD133 and CD271.^62^, ^63^, ^64^, ^65^ STAT3 activation may promote chemoresistance to OS, therapy by inhibiting the effect of chemotherapeutic agents on OSS.^63^, ^64^ Together, these findings suggest that STAT3 is a both a proto‐oncogene and molecular target of known oncogenes implicated in the development of OS. STAT3 is overexpressed in OS tissues and cell lines and is a good predictor of poor response to OS chemotherapy. Further research is needed to investigate STAT3 and its isoforms as potential targets for OS gene therapy.

### 3.2 STAT3 signalling pathways in OS

**FIGURE 4:**
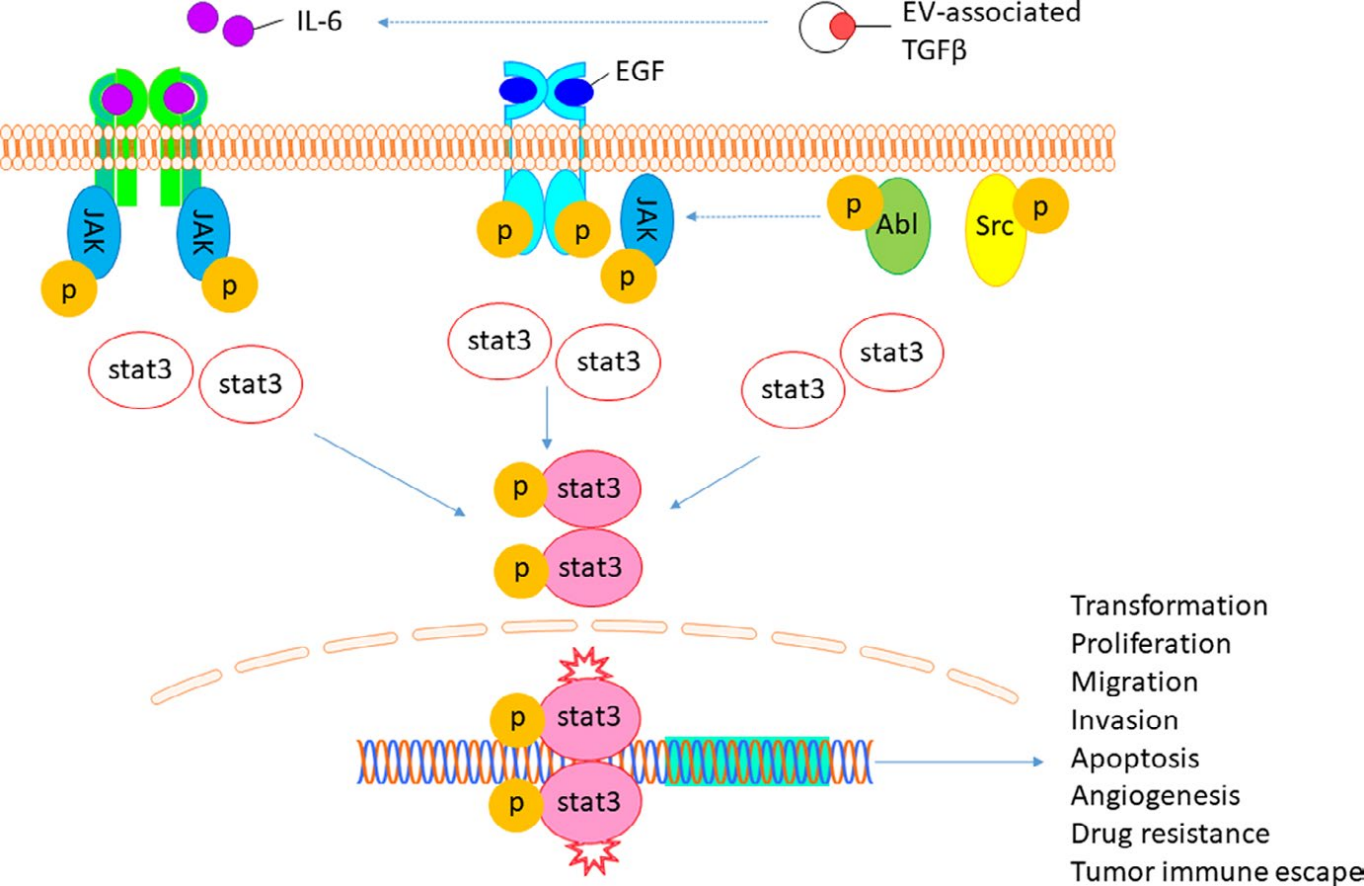
Schematic osteosarcoma cell STAT3 signalling pathways. STAT3 is activated following phosphorylation, dimer formation and nuclear translocation for the regulation of target gene transcription for processes including cellular proliferation, migration, invasion, immune evasion and drug resistance. STAT3, signal transducer and activator of transcription 3

STAT3 signalling is implicated in the development and progression of OS (Figure 4).^9^ STAT3 activation by cytokines, such as IL‐6 and LIF, has been shown to promote the growth and metastasis of OS in vitro and in vivo.^66^, ^67^ Increased IL‐6/JAK/STAT3 signalling is implicated in many human cancers and is associated with poor clinical prognosis.^68^ Src/STAT3 signalling is upregulated in OS tissues and cell lines.^69^ STAT3 activation was shown to mediate OS metastasis downstream of ΔNp63 in vitro.^70^ ΔNp63 is a splice variant of p63, which blocks the tumour suppressor activity of p53, p63 and p73.^70^ ΔNp63 was shown to upregulate IL‐6/STAT3 signalling and promote angiogenesis of OS cells in vitro.^70^ STAT3/serine/threonine kinase 35 (STK35) overexpression in OS was recently investigated.^71^ STAT3 and STK35 are overexpressed in OS on public expression and cohort data.^71^ STAT3 regulation of STK35 transcription was demonstrated by luciferase reporter assay.^71^ STAT3 signalling appears to be involved in the progression of OS via activation of the E2F1/DDR1 pathway.^72^ STAT3 overexpression pathways in OS have been demonstrated for ubiquitin‐specific protease 1, LncRNA bladder cancer associated transcript 1 and leucine‐rich repeat‐containing G protein–coupled receptor 4 in vitro.^73^, ^74^, ^75^ STAT3 overexpression in advanced OS tumours leads to decreased expression of tumour suppressor gene, PARK2.^76^ Conversely, PARK2 overexpression was shown to inhibit tumour growth and angiogenesis via the JAK2/STAT3/VEGF pathway in vivo and in vitro.^76^ Src/STAT3 signalling in OS may be deactivated by tumour suppressor effect from enamel matrix protein, ameloblastin (AMBN), representing a potential biomarker and therapeutic target for OS.^77^ Inhibition of STAT3 expression by miRNAs may be a potential avenue to identify OS biomarkers and therapeutic targets. STAT3 expression in OS was attenuated by miRNAs, miR‐199a‐3p and miR‐340‐5p, in vitro.^78^, ^79^ miR‐340‐5p overexpression resulted in decreased tumour size and weight in nude mice.^79^ Further research is needed to determine the miR‐340‐5p/STAT3 signalling potentially involved with decreased OS tumour growth in vivo. STAT3 OS target genes may include cyclin D1, Bcl‐2, Bcl‐xL, survivin and Mcl‐1, which represent potential targets for OS gene therapy.^59^, ^80^ The STAT3 signalling network involved with the progression of OS is diverse and pleiotropic. Further research is needed to identify vital STAT3 OS biomarkers and to develop potential therapeutic targets for STAT3‐directed OS gene therapy.

## 4 THE PATHOGENESIS OF STAT3 IN OS

Constitutively activated STAT3 is associated with tumorigenesis and progression of many cancers, such as haematopoietic tumours (eg, multiple myeloma and leukaemia) and solid tumours (eg, breast cancer, lung cancer and pancreatic cancer).^81^, ^82^ STAT3 is considered to be an oncogene and may regulate oncogenic events involved with cell‐cycle progression, apoptosis, tumour angiogenesis, invasion, metastasis and evasion of the immune system of OS.^82^ STAT3 signalling represents a pathogenic pathway for the growth of OS in the bone microenvironment.

### 4.1 Proliferation, migration, invasion and metastasis

Dysregulated activation of STAT3 appears to promote the proliferation of malignant tumours, including OS.^10^, ^69^, ^81^ Preclinical studies indicate increased IL‐6/JAK/STAT3 signalling in tumour cells and tumour infiltrating immune cells could promote tumour proliferation, survival, invasion and metastasis, and might suppress anti‐tumour immunity.^68^ IL‐6/STAT3 signalling in mesenchymal stem cells (MSCs) was shown to promote the proliferation, migration and metastasis of OS in vitro and in vivo.^67^, ^83^ IL‐6/STAT3 signalling upregulates the expression of genes involved with cell proliferation (c‐Myc and cyclin D1), angiogenesis (VEGF) and the inhibition of apoptosis (Bcl‐xL and survivin) in cancers.^81^ Further investigation of IL‐6/STAT3 signalling is required in OS. IL‐6/STAT3 and Oncostatin M/STAT3 signalling increased the expression of cyclin‐dependent kinase inhibitor, p21, in OS cells in vitro leading to the proliferation of OS.^84^ Constitutive activation of STAT3 downstream of Src kinases promotes the proliferation, cell‐cycle progression and survival of OS cells in vitro by the upregulation of target genes survivin, cyclin D1, Bcl‐2, Bcl‐XL, Mcl‐1, VEGF and MMP.^69^, ^80^ MMPs are overexpressed and implicated in the invasion of OS.^85^, ^86^ Epithelial‐to‐mesenchymal transition (EMT) transcription factors are involved in the pathogenesis of OS.^87^ STAT3 is a strategic target for OS drug therapy designed to inhibit the growth and development of OS by its molecular interactions with pathogenic genes, such as MMPs and EMT transcription factors. STAT3 was shown to mediate the histone deacetylase 6 (HDAC6) activation of programme death receptor ligand‐1 (PD‐L1) expression, resulting in the progression of OS in vivo due to the inhibition of T‐cell function.^88^ Research indicates that STAT3 is a potential therapeutic target for OS therapy successfully inhibiting the proliferation, migration, progression and invasion of tumour cells.^80^, ^88^, ^89^ Dysregulated STAT3 activation contributes to the development of OS, and further research is needed to develop therapeutic targets of STAT3 signalling pathways for OS treatment.

### 4.2 Angiogenesis

STAT3 is a central signalling mediator for multiple pathways, such as Src, EGF and GM‐CSF, regulating angiogenesis via the upregulation of VEGF.^90^, ^91^ Constitutive activation of STAT3 is correlated with VEGF expression in a range of cancer cell lines, including OS.^90^, ^92^ JAK2/STAT3/VEGF signalling is activated during angiogenesis in vivo, and tumour suppressor gene, PARK2, was shown to inhibit tumour growth and angiogenesis in vivo by downregulating the JAK2/STAT3/VEGF pathway.^76^, ^91^ IL‐6/STAT3 activation by splice variant ΔNp63 results in the stabilization of hypoxia‐inducible factor 1α, the secretion of VEGF and angiogenesis in OS tumours.^70^ These findings suggest that STAT3 regulates numerous pathways leading to angiogenesis in OS. Further research will aim to develop therapeutic strategies targeting STAT3 for the prevention of angiogenesis in OS.

### 4.3 Apoptosis

Dysregulated STAT3 activation has been shown to confer resistance to apoptosis in human cancers, including OS.^10^, ^55^, ^67^ Patient OS tissues have been characterized by overexpression of STAT3, metastasis and resistance to apoptosis, resulting in poor patient prognosis.^10^ IL‐6/STAT3 signalling by MSCs in the bone microenvironment led to the progression of OS and resistance to apoptosis in vivo and in vitro.^67^ IL‐6/STAT3 anti‐apoptotic activation in OS was shown to be conferred by genes cyclin D, Bcl‐xL and survivin in vitro.^67^ STAT3/STK35 signalling represents a potential anti‐apoptotic pathway of OS.^71^ STAT3 and STK35 (a target gene of STAT3) upregulation is correlated in OS tissues and mechanistically linked via the JAK/STAT pathway by gene set enrichment analysis and in vitro findings.^71^ STAT3 anti‐apoptotic target genes identified in cancers and requiring further determination specific to OS include cyclin D1, Bcl‐xL, Bcl‐2, Mcl‐1, survivin and p21.^55^, ^67^, ^93^, ^94^, ^95^, ^96^ Therapeutic inhibition of STAT3 signalling has been shown to decrease the expression of anti‐apoptotic target genes (Bcl‐xL, Mcl‐1), leading to increased expression of mitochondrial apoptosis related pathway proteins (Bax, Bak, cytosolic cytochrome c and cleaved caspase 3), which resulted in increased apoptotic index of OS cells in vitro.^97^ Disruption of STAT3 signalling in sarcoma cells, including OS, induced apoptosis by caspase‐3, caspase‐7, caspase‐8 and caspase‐9 pathways in vitro.^60^, ^69^ HDAC6/STAT3 signalling was shown to regulate PD‐L1 expression and the progression of OS in vivo, representing a potential anti‐apoptotic pathway for further investigation.^88^ Disruption of JAK2/STAT3/VEGF signalling by overexpression of PARK2 was shown to suppress OS tumour growth and angiogenesis in vivo and to induce OS apoptosis in vitro.^76^ Aberrant expression of STAT3 is a causative factor in the development, progression, metastasis and resistance to apoptosis of OS. STAT3 activation of anti‐apoptotic target genes is implicated in conferring resistance to apoptosis of OS. STAT3 anti‐apoptotic signalling pathways represent promising therapeutic targets for OS and require further investigation.^59^, ^80^

### 4.4 Autophagy

STAT3 signalling might affect the cellular process of autophagy, with subcellular localization patterns indicative of its regulatory activity.^98^ Nuclear STAT3 regulates the transcription of autophagy‐related target genes during autophagy, such as BCL2, BECN1, PIK3C3, CTSB, CTSL, PIK3R1, HIF1A and BNIP3.^98^ Nuclear STAT3 may also regulate the expression of microRNAs, which target autophagy‐related genes.^99^ Cytoplasmic STAT3 was found to inhibit autophagy by decreasing the activity of eukaryotic translation initiation factor 2‐α kinase 2 via its SH2 domain.^100^ Cytoplasmic STAT3 may also inhibit autophagy by sequestering autophagy‐related proteins, FOXO1 and FOXO3.^101^ Mitochondrial STAT3 is thought to complement nuclear and cytoplasmic STAT3, by suppressing autophagy and protecting against autophagic mitochondria degradation.^98^, ^102^, ^103^, ^104^ Mitochondrial STAT3 appears to limit oxidative stress and mitophagy by regulation of the electron transport chain and decreasing the production of reactive oxygen species.^103^ STAT3 pathways regulating autophagy may provide therapeutic targets for OS treatment, indicating the need for further research.

### 4.5 Drug resistance

STAT3 activation is attributed to both the progression and chemotherapeutic resistance of cancers, including OS.^81^ The OS tumour microenvironment is implicated in the development of drug resistance and has been investigated.^105^ Chemoresistance of OS cells appears to be influenced by MSCs within the tumour microenvironment.^105^ IL‐6/STAT3 activation was shown to regulate MSCs induction of chemoresistance in OS in vitro and in vivo.^105^ Clinical OS samples characterized by chemoresistance demonstrate high levels of p‐STAT3 and OS drug‐resistant proteins, multidrug resistance protein and p‐glycoprotein.^105^, ^106^ Moreover, OS patients with high STAT3 expression had significantly lower survival rate than those without high STAT3 levels.^105^ Inhibition of STAT3 may improve the sensitivity of chemotherapy‐resistant OS cell lines to drugs including doxorubicin and cisplatin.^77^, ^106^, ^107^ STAT3 deactivation by compounds, such as raddeanin A (RDA), represents a potential therapeutic target to increase chemosensitivity of OS cells.^106^ STAT3 inhibition appears to increase sensitivity to cisplatin by reactivating ferroptosis.^107^ OS STAT3 potential therapeutic targets to increase chemosensitivity include AMBN.^77^ Src/STAT3 inhibition by AMBN was shown to induce sensitivity to doxorubicin of OS cells.^77^ Constitutive activation of STAT3 may lead to chemoresistance of OS, failure of chemotherapy and poor prognosis for OS. STAT3 represents a potential target for OS chemotherapy via signalling pathways including IL‐6, Src and JAK2. Further research is needed to investigate STAT3‐mediated chemoresistance in OS and to develop STAT3‐targeted chemotherapeutic treatments for OS.

## 5 THE ROLE OF STAT3 IN THE OS TUMOUR MICROENVIRONMENT

STAT3 is constitutively activated in tumour cells and immune cells of the tumour microenvironment in a range of cancers, such as OS.^108^, ^109^ STAT3 is a convergent mediator of oncogenic signalling by numerous pathways, and constitutive activation of STAT3 inhibits the expression of anti‐tumour immune mediators, leading to an impaired immune response.^108^ STAT3 signalling is involved with crosstalk between tumour cells, immune cells and the microenvironment, promoting tumour‐induced immunosuppression.^108^ STAT3 activity interferes with inflammatory signals of the immune system leading to immune evasion.^108^, ^110^ Constitutive STAT3 activity appears to inhibit the anti‐tumour response by immune cells, including dendritic cells (DCs), T cells, natural killer (NK) cells and neutrophils.^109^, ^110^ STAT3 signalling between tumour cells, immune cells and the tumour microenvironment is mediated by factors, including IL‐6, IL‐10 and VEGF.^108^

### 5.1 Inflammation

Inflammation is associated with the onset and progression of cancers, including OS.^111^, ^112^, ^113^ The inflammatory response influences each stage of tumour development from initiation and promotion to malignancy, invasion and metastasis.^111^ Inflammation affects the immune cells response to cancers, immune cell interactions with cancer cells of the tumour microenvironment, and the response to cancer therapy.^111^ STAT3 signalling activated by inflammatory factors, such as IL‐6, cyclooxygenase‐2 (COX‐2) and TGF‐β, appears to be involved in the development of OS.^67^, ^114^, ^115^ Inflammatory factors may be secreted by cells of the tumour microenvironment, such as MSCs and macrophages.^67^, ^114^ Inflammatory cytokine IL‐6/STAT3 signalling from MSCs in the tumour microenvironment appears to promote the survival, proliferation, metastasis and drug resistance of OS.^67^, ^105^ Further, constitutive TGFβ/IL‐6/STAT3 activation, tumour growth and lung metastasis in OS is perpetuated by paracrine activity from tumour extracellular vesicle–educated MSCs (TEMSCs) in mouse and human OS tissue samples.^115^ Inflammatory COX‐2/STAT3 signalling is upregulated by tumour‐associated macrophages (TAMs) and promotes OS cell migration, invasion, and EMT in mouse and OS patients.^114^ Together, these findings indicate that STAT3‐mediated inflammation influences the OS tumour microenvironment at each stage of tumour development leading to the progression, metastasis and drug resistance of OS. Cells from the tumour microenvironment, such as TEMSCs and TAMs, are potential therapeutic targets for OS therapy involving STAT3 signalling pathways.

### 5.2 Immune evasion by OS tumours

STAT3 activity appears to mediate immune evasion by tumours, such as OS, by suppressing the tumour‐specific expression of pro‐inflammatory mediators.^110^ Constitutive STAT3 activity in tumours increases the production of pleiotropic factors, such as IL‐10 and VEGF, which inhibit DC maturation via STAT3 signalling.^110^ STAT3 inhibition in tumour cells resulted in the production of pro‐inflammatory cytokines and chemokines, leading to activation of the anti‐tumour innate immune response, DCs activation and tumour‐specific T‐cell response.^110^ STAT3 ablation in tumour cells leads to improved immune function of DCs, NK cells, T cells and neutrophils, and tumour regression in vivo.^109^ Constitutive STAT3 and mitogen‐activated protein kinase (MAPK) signalling is involved with tumour immune evasion of human melanomas.^116^ Validation of these findings is necessary in the context of OS. The role of STAT3 in mediating immune evasion by OS tumours is largely unknown. STAT3/COX‐2 signalling is thought to regulate immunosuppression by myeloid‐derived suppressor cells in OS.^117^ Immune evasion by metastatic OS, but not primary tumours, was shown to be mediated by the interaction between programmed death receptor‐1 (PD‐1) on cytotoxic T‐lymphocytes (CTLs) and its ligand, PD‐L1, on tumour cells, due to inhibition of CTL function in vivo.^118^ The effect of STAT3 signalling in regulating PD‐1 expression in OS T‐cell populations is unknown and requires further investigation. Further research is required to determine the role of STAT3 in mediating immune evasion in OS.

## 6 STAT3 INHIBITORS IN OS TREATMENT

OS is a primary malignant bone tumour exhibiting aggressive growth and metastasis, leading to high mortality. The five‐year survival rate of metastatic OS is estimated to be 20%‐30% and has not improved significantly in the previous decades.^119^, ^120^ Targeted therapy for primary OS, metastatic OS and MDR OS to improve the prognosis of OS treatment is an intensive focus of research. STAT3 is a prime therapeutic target for OS treatment, and STAT3 inhibition appears to be a promising therapy for OS.^89^, ^121^ STAT3 inhibitors may be obtained from natural or synthetic sources and divided into two categories including direct and indirect inhibitors.^81^, ^122^, ^123^ Pharmacological inhibition of STAT3 activity by tyrosine kinase inhibitors, anti‐sense oligonucleotides, dominant negative proteins, RNA interference and chemo‐preventive compounds represent potential strategies of STAT3‐directed OS therapy. Direct STAT3 inhibitors may prevent oncogenic transcription by blocking STAT3 dimerization and/or DNA binding.^124^ Indirect STAT3 inhibitors block upstream activators of STAT3, such as cytokines IL‐6 and IL‐10, or growth factors, such as VEGF and EGF, and are potential suppressors of the STAT3‐signalling cascade of OS.^125^ Targeting components of the IL‐6/JAK/STAT3 signalling pathway is a potential therapeutic approach, with registered clinical trials of several STAT3 inhibitors.^68^ STAT3‐coordinated signalling circuits are crucial for the development of cancers, such as OS, and represent therapeutic targets that require further investigation.^126^ STAT3 is an intracellular transcription factor, and a difficult target for drug therapy.^127^ The identification and development of novel and potent STAT3 inhibitors for OS therapy is a challenging area of research.

### 6.1 OS therapeutic treatment

#### 6.1.1 Synthetic inhibitors

Synthetic pharmacological agents play a leading role in the search for safe and effective chemotherapeutic cancer treatments. Synthetic analogues of naturally occurring compounds with anti‐tumour properties may be designed in order to deliver the level of bioactivity required for chemotherapeutic effect.^128^ Structural analogues of established anti‐cancer drugs may be designed for OS therapy.^129^ Chemical libraries may be utilized to identify and design STAT3 inhibitors with therapeutic potential for the treatment of OS.^130^ Synthetic small molecule STAT3 inhibitors are potentially effective for the treatment of OS and may inhibit STAT3‐signalling directly or indirectly.^131^, ^132^, ^133^ Current research is dedicated to determining novel chemotherapeutic agents with potential for successful OS treatment.

##### Direct Inhibitors of STAT3

Direct inhibition of STAT3 activation may include blocking the phosphorylation of STAT3, preventing STAT3 dimerization and nuclear translocation, and disrupting DNA binding by STAT3 in the nucleus. Direct STAT3 inhibitors are designed to target structural elements of the STAT3 protein important for its function, such as the SH2 domain, N‐terminal domain and C‐terminus transcriptional activation domain.^123^ S3I‐201 (NSC 74859) was identified from the National Cancer Institute chemical libraries as an inhibitor of STAT3 homodimer formation, DNA binding and transcriptional activities.^130^ S3I‐201 targets the SH2 domain of STAT3 and was shown to inhibit the growth and induce apoptosis of breast tumour cells in vivo, by downregulating the expression of STAT3‐target genes, cyclin D1, Bcl‐xL and surviving.^130^ S3I‐201 shows potential for the treatment of cancers, including breast and liver cancer, and requires further investigation and development for OS treatment.^134^ Small molecule, S3I‐1757, prevents STAT3 dimerization and activation by targeting Y705 of the SH2 domain.^135^ S3I‐1757 was shown to inhibit STAT3 DNA binding and transcriptional activation of target genes, Bcl‐xL, cyclin D1, survivin and MMP‐9, leading to anti‐cancer activity in vitro.^135^ S3I‐1757 demonstrates the potential to inhibit the growth and metastatic transformation of cancer cells and requires further investigation of its potential for the treatment of OS. BP‐1‐102 was designed by computer‐aided optimization and inhibits STAT3 activation and function.^136^ BP‐1‐102 is potentially effective for the treatment of breast cancer and by inhibiting the growth and metastatic transformation of cancer cells in vivo.^136^ BP‐1‐102 demonstrated the ability to regress lung cancer xenografts and further evaluation of its potential for OS treatment is required.^136^ BP‐1‐102 is orally bioavailable in preclinical models.^68^ FLLL32 is a structural analogue of naturally occurring curcumin, designed to specifically target and interfere with STAT3 function in cancers, including OS.^128^, ^137^ FLLL32 was shown to inhibit STAT3 DNA binding and target gene transcription, and to induce apoptosis in OS cell lines.^128^ Small non‐peptide molecule, LLL12, was designed to directly inhibit STAT3 activation.^138^ LLL12 was shown to prevent phosphorylation of STAT3 at Y705, and to induce apoptosis in cancer cells.^138^ LLL12 inhibited STAT3 DNA binding and decreased the expression of STAT3 target genes implicated in oncogenesis, specifically survivin, cyclin D1 and Bcl‐2.^138^ LLL12 and FLLL32 have been shown to inhibit STAT3 directly and indirectly via IL‐6‐mediated phosphorylation in human rhabdomyosarcoma cells, OS cells and a murine model of OS.^139^, ^140^ LLL12 was shown to enhance the anti‐proliferative chemotherapeutic effect of doxorubicin against OS cell lines, and requires further investigation of its potential for combined drug therapy for OS treatment.^141^ SC‐1, a structural analogue of cancer drug, sorafenib, was shown to inhibit OS cell proliferation and tumour growth in vivo.^129^ SC‐1 decreases STAT3 activation by directly targeting Y705 phosphorylation, and disrupting JAK/STAT3 signalling in an SH2‐dependent manner.^129^ Synthetic oleanane triterpenoid, C‐28 methyl ester of 2‐cyano‐3,12‐dioxoolen‐1,9‐dien‐28‐oic acid (CDDO‐Me) effectively blocks STAT3 phosphorylation and inhibits STAT3 nuclear translocation, resulting in apoptosis of OS cell lines.^59^ CDDO‐Me appears to be effective against MDR OS cells and tissues by decreasing the expression of anti‐apoptotic target genes, Bcl‐xL, survivin and MCL‐1.^59^ CDDO‐Me enhanced the cytotoxic effect of doxorubicin against MDR OS cells, representing a potential combined therapeutic approach for the treatment of OS.^59^ CDDO‐Me appears to be a promising drug for OS therapy and requires further development of its clinical potential.^59^ Small molecule inhibitor, LY5, was designed to prevent STAT3 homodimer formation by blocking the SH2 domain phosphotyrosine‐binding site.^142^ LY5 was shown to mediate anti‐cancer effects on OS cells in vitro.^142^ Further research is necessary to determine the signalling pathways mediating the anti‐cancer effects of LY5. Anti‐psychotic drug, pimozide, appears to be a novel STAT3 inhibitor with potential for OS treatment and requires further investigation.^143^ Together, these findings suggest that direct STAT3 inhibitors are potentially effective for the treatment of OS and require further research to develop their clinical application.

##### Indirect inhibitors of STAT3

Indirect inhibition of STAT3 may be achieved by blocking upstream regulators of STAT3‐signalling, such as IL‐6 and EGF.^125^ Inhibition of JAK/STAT signalling is an established strategy of targeting STAT3 in cancer therapy.^144^ Tyrphostin AG490 is a Jak tyrosine kinase inhibitor and upstream inhibitor of STAT3 with therapeutic potential for OS.^145^, ^146^ AG490 has been shown to suppress the growth and induce apoptosis of IL‐6‐dependent multiple myeloma cells by downregulating the STAT3 and MAPK signalling pathways.^145^ AG490 may be effective for the treatment of OS when combined with drugs, such as pterostilbene, and requires further investigation for the potential treatment of OS.^97^ INCB018424 and cepharanthine (CEP)‐701 are selective JAK inhibitors, which have been shown to suppress IL‐6/STAT3 signalling, and have potential for the treatment of cancers, such as OS.^125^, ^144^, ^147^ Irisin was identified as a hormone produced during exercise and it appears to have potential for the treatment of OS.^148^, ^149^ Irisin treatment demonstrated the reversal of IL‐6 induced EMT in OS cells and inhibited the proliferation, migration and invasion of OS cells in vitro.^148^ Irisin suppressed the IL‐6 activation of STAT3 via the STAT3/Snail signalling pathway.^148^ BBI608, or napabucasin, is a small molecule identified by its ability to inhibit STAT3‐regulated transcription and cancer stemness.^150^ Napabucasin (BBI608) treatment induced apoptosis of OS cells in vitro and inhibited OS tumour growth and metastasis in an in vivo OS model.^121^ Further investigation of the molecular mechanism and signalling pathway by which BBI608 inhibits STAT3 activity and OS progression is required. YN968D1, or apatinib, is a small molecule inhibitor of VEGF signalling.^151^ Apatinib inhibits the kinase activity of VEGFR‐2, c‐Src, c‐Kit and PDGFRβ.^151^ Apatinib was shown to inhibit the growth and induce apoptosis of OS cells in vitro, and to suppress the growth of OS in vivo via disruption of VEGFR/STAT3/BCL‐2 signalling.^152^ Novel heat shock protein 90 (HSP90) inhibitor, STA‐1474, inhibited the proliferation and induced apoptosis of OS cell lines and inhibited the growth of OS tumours in vivo.^131^ STA‐1474 downregulated the expression of p‐Met/Met, p‐Akt/Akt and p‐STAT3 and warrants further investigation as a potential chemotherapeutic agent for the treatment of OS.^131^ Together, these findings indicate there are numerous pharmacologic agents with potential chemotherapeutic application for the treatment of OS. Further research is necessary to develop promising OS therapies indirectly targeting STAT3‐signalling pathways.

#### 6.1.2 Natural compounds

Natural compounds are potentially effective for the treatment of OS and may be developed as synthetic analogues for OS therapy. Natural compounds present an alternative to chemotherapy using agents, such as cisplatin, doxorubicin and methotrexate, and may offer hope to sufferers of OS with poor prognosis. Natural compounds appear to target signalling pathways involved in OS, such as JAK/STAT, PI3K/AKT, Notch and Wnt.^153^ Natural compounds, including curcumin, diallyl trisulfide, resveratrol, apigenin, cyclopamine and sulforaphane may be grouped by their chemical structures and have potential therapeutic application in the treatment of OS. Here, we focus on natural compounds currently thought to inhibit STAT3‐signalling in the context of OS therapy.

##### Polyphenolic

Resveratrol is a natural polyphenolic compound, which may have potential for the treatment of OS.^64^ Resveratrol was shown to inhibit OS cell viability and tumour growth in vitro via downregulation of the JAK2/STAT pathway.^64^ Pterostilbene, a natural analogue of resveratrol with higher bioavailability, appears to be a potent inhibitor of OS cell growth via disruption of JAK2/STAT signalling, and its anti‐OS effects may be enhanced when used in combination with AG490.^97^ Pterostilbene has the potential to induce apoptosis of OS cells and requires further investigation for OS therapy.^97^ Curcumin was shown to inhibit the proliferation and migration of OS cells via JAK2/STAT signalling, and synthetic curcumin analogue, FLLL32, is a potential anti‐OS drug.^128^, ^154^ Chlorogenic acid is a polyphenol compound and was shown to inhibit OS cell growth and induce apoptosis in vitro via the STAT3/Snail pathway.^155^ Further research is required to advance the therapeutic potential of polyphenolic compounds for OS.

##### Flavonoids

Flavonoids, such as ginkgetin, appear to have potential for the treatment of OS.^156^ Ginkgetin, a biflavone extracted from the leaves of ginkgo biloba, was found to inhibit the growth and activate apoptosis of OS cells in a concentration‐dependent manner via decreased STAT3 expression and activation of caspase‐3/9.^156^

##### Alkaloids

Alkaloids, such as CEP, coptisine, sinomenine and columbamine, appear to have therapeutic potential against OS via inhibition of STAT3‐signalling pathways, and require further investigation.^157^, ^158^, ^159^, ^160^ Sinomenine was shown to inhibit OS cell invasion and metastasis by downregulating CXCR4/STAT3 signalling, resulting in decreased expression of OS target genes, MMP‐2 and ‐9, RANKL and VEGF.^159^ Sinomenine was shown to reduce OS progression and metastasis in vivo and appears to be a promising therapeutic agent for OS treatment.^159^

##### Terpenoid

Terpenoids, such as toosendanin, cucurbitacin B and I, RDA, glaucocalyxin A and catalpol, appear to have anti‐OS potential via modulation of STAT3 signalling.^161^, ^162^, ^163^ Toosendanin appears to inhibit OS tumour progression by preventing STAT3 dimerization and blocking STAT3/EGFR signalling.^161^ Cucurbitacin B and I are novel STAT3 inhibitors with potential for OS treatment and require further investigation.^164^, ^165^ RDA was shown to inhibit OS cell growth and promote OS cell apoptosis in vivo by disruption of IL‐6/JAK2/STAT3 signalling.^106^ RDA may present a potential treatment option for doxorubicin resistance in OS.^106^ Together, terpenoids require further research for their potential use in OS therapy.

##### Quinones

4‐methoxydalbergione (4‐MD), extracted from Dalbergia odorifera, belongs to the quinones group of compounds and possesses anti‐oxidant, anti‐inflammatory, as well as anti‐tumour properties. 4‐MD demonstrated the inhibition of OS cell proliferation and induced OS cell apoptosis in vitro by downregulating JAK2/STAT3 signalling. 4‐MD inhibited OS tumour growth in a murine xenograft in vivo model by decreasing the expression of STAT3 and anti‐apoptosis gene, surviving.^166^ Therefore, 4‐MD may be a potential anti‐OS therapeutic quinone compound and requires further investigation.

## 7 CONCLUSIONS AND FUTURE PERSPECTIVES

STAT3 is a cytoplasmic transcription factor of the STAT family and is activated by tyrosine phosphorylation by numerous signalling pathways. STAT3 is activated by receptor tyrosine kinases, including cytokines of the IL‐6 family signalling by the gp130 subunit, and growth factors, such as EGF, and non‐receptor tyrosine kinases, such as Src and Abl. STAT3 may also be activated as a downstream effector of G protein signalling. STAT3 is a convergent intracellular signalling mediator for a range of pathways. Activated STAT3 is translocated to the nucleus for the transcriptional regulation of target genes involved with vital biological processes, including embryogenesis, immunity, haematopoiesis and apoptosis. The activation of STAT3 is rapid, transient and tightly regulated under physiological conditions. Four STAT3 isoforms have been identified, which have distinct biological functions. The role of STAT3 isoforms in cancers, including OS, is largely unknown and requires further investigation. Constitutive activation of STAT3 appears to lead to the onset and progression of OS. STAT3 is considered a proto‐oncogene, and evidence suggests that dysregulated expression of STAT3 plays an oncogenic role in OS by promoting processes including cellular transformation, tumour growth, invasion, metastasis, resistance to chemotherapy and immune evasion. Oncogenic STAT3 target genes include cyclin D1, Bcl‐xL, survivin, Mcl‐1 and MMPs. STAT3 is a potential target for OS therapy, and STAT3 inhibitors appear to be potentially effective for the treatment of OS. STAT3 inhibitors may act directly or indirectly to downregulate the expression of target genes involved with OS. Further research is needed to develop the therapeutic potential of STAT3 inhibitors from synthetic sources and natural compounds, including possibilities for improved chemotherapeutic efficacy by a combined drug approach.

## CONFLICT OF INTEREST

All authors declare that they have no conflict of interest.

## AUTHOR CONTRIBUTIONS

Yun Liu and Shijie Liao conducted research and drafted the manuscript. Samuel Bennett, Haijun Tang, and Dezhi Song aided in the process of drafting manuscript and protein structural analysis. Jiake Xu performed the bioinformatics analysis of gene expression. David Wood, Xinli Zhan^,^ and Jiake Xu supervised the study and revised the manuscript.

## Data Availability

The data that support the findings of this study are available from the corresponding author upon reasonable request.
